# CDX2 as a Predictive Biomarker Involved in Immunotherapy Response Suppresses Metastasis through EMT in Colorectal Cancer

**DOI:** 10.1155/2022/9025668

**Published:** 2022-10-12

**Authors:** Yun-shuai Wang, Yu Kou, Ren-tao Zhu, Bao-wei Han, Chao-hui Li, Huang-jian Wang, Hui-bin Wu, Tian-ming Xia, Xiang-ming Che

**Affiliations:** ^1^Department of General Surgery, The First Affiliated Hospital of Xi'an Jiaotong University, Xi'an, 710000 Shanxi, China; ^2^Department of General Surgery, Luoyang Central Hospital Affiliated of Zhengzhou University, Luoyang, 471000 Henan, China; ^3^Institute of Translational Medicine, Medical College, Yangzhou University, Yangzhou, 225000 Jiangsu, China; ^4^The Key Laboratory of Syndrome Differentiation and Treatment of Gastric Cancer of the State Administration of Traditional Chinese Medicine, Yangzhou, 225000 Jiangsu, China; ^5^Jiangsu Key Laboratory of Integrated Traditional Chinese and Western Medicine for Prevention and Treatment of Senile Diseases, Medical College of Yangzhou University, Yangzhou, 225000 Jiangsu, China

## Abstract

**Background:**

Studies have confirmed that Caudal Type Homeobox 2 (CDX2) plays a tumor suppressor role in colorectal cancer (CRC) and as a prognostic and predictive marker for colorectal cancer. The epithelial to mesenchymal transition (EMT) is a transdifferentiation process, providing migratory and invasive properties to cancer cells during tumor progression. However, the role of CDX2 during the activation of EMT in CRC maintains controversial.

**Aim:**

To investigate whether CDX2 is associated with EMT in CRC.

**Methods:**

Forty-six CRC patients were included in the study. Expressions of CDX2, E-cadherin, and N-cadherin in all CRC patients were detected by IHC. ROC assays were applied to detect cut-off points for IHC scores to distinguish high and low expressions of CDX2 in 46 CRC samples. The prognostic value of CDX2 was statistically analyzed. MTT, Western blot, invasion, and migration assays in vitro were employed to explore the function of CDX2.

**Results:**

We observed that high expressions of CDX2 and E-cadherin as well as low expressions of N-cadherin were significantly correlated with favorable prognosis. The levels of CDX2 protein exhibited a positive associated with E-cadherin while negative correlation with N-cadherin. Then, the low expression of CDX2 and high expression of CA199 in combination are positively related with poor prognosis. Overexpression of CDX2 reduced expression of MMP-2 and diminished cell proliferation, invasion, and migration, while knockdown CDX2 enhanced MMP-2 expression and increased cell proliferation, invasion, and migration in HCT-116 cells. CDX2 was correlated with expression of EMT markers. Overexpression of CDX2 suppressed the EMT markers indicating that CDX2 suppresses CRC cell viability, invasion, and metastasis through inhibiting EMT. Finally, we found that the expression of CDX2 was negatively associated with Th1 cells, macrophages, Th2 cells, cytotoxic cells, T cells, and T helper cells.

**Conclusions:**

These results indicated CDX2 as prognostic biomarkers involved in immunotherapy response for CRC. CDX2 loss promotes metastasis in CRC through a CDX2-dependent mechanism.

## 1. Introduction

Worldwide, 1.8 million new patients are diagnosed as colorectal cancer (CRC) each year, with five-year survival rate of about 50% [1]. The tumor node metastasis (TNM) classification system offers the highlight clinical framework to evaluate CRC prognosis, and united with a few molecular markers and clinicopathological characteristics, it forms the conventional basis to assess prognosis. There are a number of different serum markers that have been used as indicators for CRC diagnosis, postoperative monitoring, and surveillance of treatment effects. These include glycoprotein carcinoembryonic antigen (CEA), cancer antigen 19-9 (CA19-9), cancer antigen 72-4 (CA72-4), and cancer antigen 125 (CA125) [2, 3]. Although numbers of progress have been accomplished in early examination and multimodality treatment of CRC, patients with advanced CRC have a bad prognosis in most cases [1, 4]. Relapse and metastasis are the principal reason of death for CRC patients [5, 6]. The discovery of underlying strong biomarkers that indicate patients with a high risk of recurrence, who might focus improved therapy methods for improving the prognosis of patients with CRC, is an important step toward achieving this goal.

Caudal Type Homeobox 2 (CDX2) is a homeobox gene known as a transcription factor which has been playing a crucial role in differentiation of epithelial cells and in the development of small, midgut, hindgut, and large intestine in mammals [7, 8]. Dalerba et al. found that CDX2 is a prognostic factor and emerging biomarker in CRC [9]. Then, CDX2 is a comparatively sensitive and particular intestinal marker; hence, it is currently used to diagnose CRC clinically. Recently, Olsen et al. supported that CDX2 play a crucial role in tumor suppressor during CRC [10]. Several studies have shown that an absence of CDX2 expression level is negatively associated with tumor grade, excellent differentiation, and a favorable patient prognosis. This absence of CDX2 expression level occurs in less than thirty percent of human CRC cases [10, 11]. Moreover, loss of CDX2 expressions was considered as predictive biomarker and a poor prognostic for the response to chemotherapy in stages II [9] and III CRC [12]. Recent research has shown that the absence of CDX2 is an independent negative prognostic marker in patients with metastatic CRC who have undergone curative liver metastasis resection. This finding suggests that CDX2 loss could be used as a potential biomarker to identify patients who will only have limited benefit from surgery [13]. During EMT induction, the downregulation of CDX2 caused by EGF/bFGF is responsible for the promotion of sLex/a expression through the transcriptional suppression of FUT3 [14]. However, CDX2 can work together with beta-catenin to regulate tight junctions via promoting the expressions of claudin-1, which leads to an increase in invasion and EMT in colorectal cancer cells [15]. As a result, the functions of CDX2 throughout the process of EMT activation in CRC remained a contentious issue.

The EMT is a transdifferentiation process. During this process, the cells lose their polarity and connect with neighboring cells. Subsequently, the cells obtain mesenchymal-like and motile phenotypes. This process can be ectopically reactivated in diseases such as cancer, giving the cancer the ability to invade other tissues and spread [16]. During the growth of a tumor, EMT endows cancer cells with the ability to migrate and invade surrounding tissue [16, 17]. During the EMT step, CRC cells express affluent mesenchymal markers such as N-cadherin and Vimentin, reducing their expression of cellular adhesion proteins such as E-cadherin. Generally, decrease of E-cadherin expression level is deemed as a hallmark of the EMT process [18, 19]. The EMT is initiated by the momentary activation of a number of different oncogenic signaling pathways, which induces the reversible activation of transcription factors such as Slug, Twist, Snail, and ZEB family members [20]. In addition, SNAIL and ZEB2 are responsible for the stimulation of the expressions of matrix metalloproteinases (MMPs), which are responsible for the breakdown of the basement membrane and the facilitation of cell invasion [21, 22]. Clinically, EMT is related to a poor outcome [23].

In the present study, we used tissue immunohistochemistry to investigate the expression of CDX2 and EMT markers in CRC, and we also looked at how these indicators were associated with one another. In addition, in order to shed light on the roles that these factors play in CRC prognosis, we investigated the correlations between the expressions of CDX2 and EMT markers, as well as the levels of CA199 and CEA, and pathological characteristics and clinical outcomes. In addition, we found a link between the expression of CDX2 and the processes of proliferation, invasion, metastasis, and EMT in vitro.

## 2. Materials and Methods

### 2.1. Patients and Specimens

In order to investigate the clinical significances of CDX2, E-cadherin, and N-cadherin, we collected 46 patients from CRC patients who were undergoing therapy at the Luoyang Central Hospital Affiliated to Zhengzhou University (Luoyang, China) between May 2014 and June 2016. These patients had been diagnosed with colorectal carcinoma based on clinical and histopathological evidence. No preoperative treatment was given to any of the patients, and all of them subsequently underwent adjuvant chemotherapy after their operations. The Institutional Review Board and the Human Ethics Committee at Luoyang Central Hospital, which is affiliated with Zhengzhou University, gave their blessing to this research. In addition, every patient supplied both a written and verbal consent form. For the purpose of the immunohistochemistry (IHC) study, a total of 46 CRC samples were utilized.

### 2.2. Immunohistochemistry

On surgical tissue specimens that had previously been formalin-fixed and paraffin-embedded, immunohistochemical staining was performed. The thickness of the slides was trimmed to be 4 micrometers. Following dewaxing in xylene, paraffin sections were rehydrated in a series of ethanol solutions that gradually became more concentrated. After 10 minutes of treatment with 3% hydrogen peroxide to inhibit endogenous peroxidases, the sample was fixed in 4% paraformaldehyde for an additional 15 minutes. Sections were rinsed with PBS three times for 5 min. After that, antigen retrieval was carried out in citrate buffer (0.01 M) for three minutes at a temperature of 95 degrees Celsius using a microwave oven. Slides were then incubated with primary antibodies against CDX2 (Cat# 12306S, Cell Signaling Technology, 1 : 500), E-cadherin (Cat# 14472S, Cell Signaling Technology, 1 : 500), or N-cadherin (Cat# 13116S, Cell Signaling Technology, 1 : 500) overnight at 4°C, followed by treatment with biotinylated secondary antibodies for 30 min at room temperature and then streptavidin-biotin complex (SABC, Boster). Slides were digitally photographed with equal light exposure in order to quantify the immunostaining for CDX2, E-cadherin, and N-cadherin. This analysis was performed using Image-Pro Plus (IPP). The immunostaining extent was rated on a scale of 0 to 100 based on the proportion of positively stained cells.

### 2.3. Cell Culture

The human CRC HCT-116 cell line was provided by the Cell Bank at the Chinese Academy of Sciences (Shanghai, China). The HCT-116 cells were kept alive in a mixture consisting of DMEM (BI, Israel) supplemented with 10% FBS (BI, Israel) at a temperature of 37 degrees Celsius and 5% carbon dioxide. After growing the cells in culture until they reached 85% confluence, they were passed through the lab via trypsinization.

### 2.4. Lentiviral Transduction

Green fluorescent protein (GFP) and a puromycin resistance gene were carried by lentiviral vectors that were used for CDX2 overexpression and knockdown, respectively. In the presence of a multiplicity of infection (MOI) of 10 and at a concentration of 10 g/ml polybrene, cells were transduced with the appropriate lentiviral vector encoding the gene of interest (Sigma-Aldrich, St. Louis, MO, USA). In addition, in order to control for the effects of viral vector transduction, each cell line was transduced with a nontargeting negative control lentiviral vector using the same method. After incubation for 12 hours at 37 degrees Celsius, the medium was changed out for a fresh batch of the suitable media. After incubation for forty-eight hours, a concentration of two micrograms per milliliter of puromycin (Sigma-Aldrich) was applied in order to select for stably transduced cell lines. Evaluation of transduction efficiency was performed 72 hours after transduction by counting GFP positive cells using a fluorescence microscope.

### 2.5. MTT (3-(4,5-Dimethylthiazol-2-yl)-2,5-Diphenyl Tetrazolium Bromide)

HCT-116 cells were seeded into 96-well plates at a density of 1.0 × 10^4^ cells per well. After exposing the cells to the MTT reagent at a concentration of 0.5 mg/ml for a period of four hours, the formazan was subsequently dissolved in dimethyl sulfoxide (DMSO). A microplate reader (PerkinElmer, Massachusetts, United States) was used to determine the OD value at 490 nm.

### 2.6. Transwell and Wound Healing Assays

Cell invasion experiments were performed using 24-well transwell that were precoated with Matrigel and had 8-micrometer pores. Seeding of HCT-116 cells at a density of 1.5 × 104 cells per well was performed in the upper chamber, which contained 1% FBS; the lower chamber contained 15% FBS. Following a period of incubation lasting for forty-eight hours, the Matrigel and the cells were removed with cotton swabs. Cells that had traversed the surface of the membrane were fixed in paraformaldehyde at a concentration of 4% and stained with crystal violet at a concentration of 0.1%. The crystal violet was then dissolved in DMSO, and the optical density was measured using an EnSpire Multilabel Reader from PerkinElmer in Massachusetts, USA, at a wavelength of 600 nm. In order to conduct a wound healing assay, HCT-116 cells were seeded in 6-well plates until they reached 95% confluency. Following this step, the plates were scraped in the central location, and images were viewed under a microscope at 0, 24, and 48 hours.

### 2.7. Western Blot Analysis

Cells that had been harvested were lysed in RIPA buffer that included 1% PMSF. SDS-PAGE was applied to separate 30 g of total proteins from each sample, and the separated proteins were then transferred to a nitrocellulose membrane. Primary antibodies included CDX2 (Cat# 12306S, Cell Signaling Technology, 1 : 1000), E-cadherin (Cat# 14472S, Cell Signaling Technology, 1 : 1000), N-cadherin (Cat# 13116S, Cell Signaling Technology, 1 : 1000), Vimentin (Cat# ARG66199, 1 : 1000), Snail (Cat# 3879S, Cell Signaling Technology, 1 : 1000), and MMP2 (Cat# 40994S, Cell Signaling Technology, 1 : 1000). The densities of bands were measured by ECL chemiluminescence (Bio-Rad, USA).

### 2.8. Statistical Analyses

The Pearson coefficient and the *P* value were used to measure the relativity analyses that were conducted between CDX2, E-cadherin, and N-cadherin. Analyses of the receiver operating curve (ROC) were carried out in order to determine the cut points of the immunohistochemical (IHC) scores for CDX2, E-cadherin, and N-cadherin in CRC samples. The chi-square test was utilized in order to evaluate the degree of correlation that existed between CDX2, E-cadherin, and N-cadherin, in addition to clinicopathological features. In order to determine differences in survival rates and prognostic factors, the Kaplan-Meier method in conjunction with the log-rank test was used. Only the covariates that had a *P* value of less than 0.05 in the log-rank univariate analysis were incorporated into Cox's proportional hazard model for the multivariate regression. The hazard ratio (HR) and its associated 95% confidence interval were used to estimate the survival outcomes. Statistical analysis was performed using SPSS 20.0 and GraphPad Prism 5.0 (GraphPad Software, San Diego, CA, USA) software. A *P* < 0.05 was considered statistically significant.

## 3. Results

### 3.1. CRC Patients with CDX2 High Have a Very Good Prognosis

**Figure 1:**
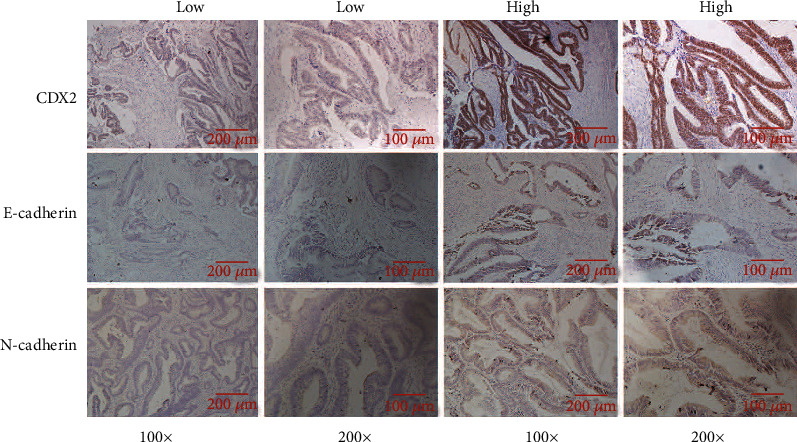
Detection of CDX2, E-cadherin, and N-cadherin using HE in colorectal cancer tissues.

**Figure 2:**
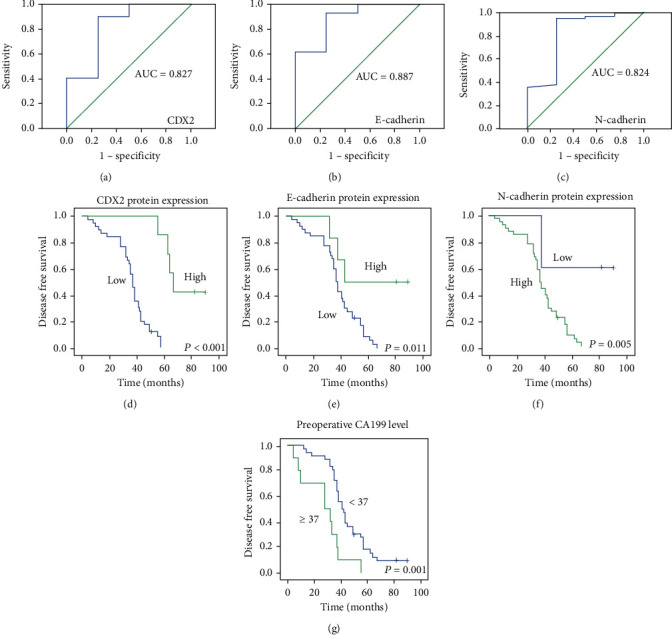
Aberrant CDX2, E-cadherin, and N-cadherin illustrate the outcomes in CRC patients for RFS. (a) CDX2, (b) E-cadherin, and (c) N-cadherin in CRC samples. (d, e) High expressions of CDX2 and E-cadherin were related to favorable outcomes in CRC samples. (f, g) High expressions of N-cadherin and CA199 are correlated with poor prognosis in human colorectal cancer samples.

**Figure 3:**
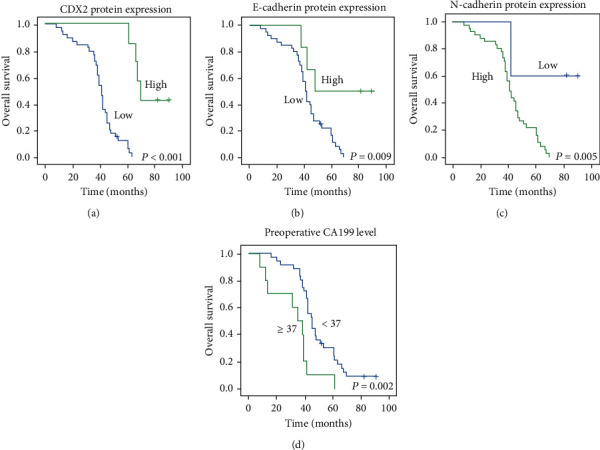
Aberrant CDX2, E-cadherin, and N-cadherin expressions illustrated the outcomes in CRC patients for OS. (a, b) Cumulative OS differences between patients with high level of CDX2 and E-cadherin. (c, d) High expressions of N-cadherin and CA199 are correlated with poor prognosis in CRC samples for OS.

To study whether the expression of CDX2 has differences in CRC, we measured the expression of CDX2 using immunohistochemical (Figure 1). The research consists of 60 patients with CRC. ROC assays were applied to detect cut-off points for IHC scores to distinguish high and low expressions of CDX2 in 46 CRC samples (Figures 2(a)–2(c)). The IHC scores of CDX2 ≥ 67.58 were regarded as high expression. CDX2 staining was successful for 46 patients: 39 (84.9%) had low expression level and 7 (15.1%) had high expression level. The Kaplan-Meier analysis demonstrates that patients with CDX2 low-expressing tumors had remarkably lower DFS and OS than those with CDX2 high-expressing tumors (log-rank test, *P* < 0.05) (Figures 2(d) and 3(a)). In the univariate Cox regression analysis, our research found that patients with CDX2-low expression had a remarkably shorter DFS and OS than patients with CDX2-high expression (both *P* < 0.001). In the multivariate Cox regression analysis, we discovered that CDX2-low was an independent adverse prognostic marker: DFS 28.068 (3.699-212.960) (Table S1) and OS 38.902 (4.979-303.962) (both *P* < 0.05) (Table S2).

### 3.2. Relationship among the Expression of CDX2, Pathological Features, and Stage of CRC

On further assessment of the correlations among CDX2 and clinical pathological parameters, we suggested that CDX2 was meaningfully related to tumor size (<3 cm and ≥3; *P* < 0.05), depth grading of tumor invasion (T1+T2 and T3+T4; *P* < 0.05), and lymph node status (N0 and N1 + N2; *P* < 0.01) (Table S3).

### 3.3. CRC Patients with E-Cadherin Low and N-Cadherin High Have a Very Poor Prognosis

ROC assays were applied to test cut-off points for IHC scores to distinguish high and low expressions of E-cadherin and N-cadherin in 46 CRC samples. The IHC scores of E − cadherin ≥ 133.1 and N − cadherin ≥ 25.66 were considered as high expression (Figure 1). E-cadherin status revealed 5-year DFS and OS rates of 50% and 50% in the E-cadherin high group and 7.5% and 20% in the E-cadherin low group, respectively (Figures 2(e) and 3(b)). Then, N-cadherin status revealed 5-year DFS and OS rates of 7.32% and 19.5% in the N-cadherin high group and 60% and 60% in the N-cadherin low group, respectively (Figures 2(f) and 3(c)). The univariate analysis revealed that E-cadherin low cases had a worse DFS and OS (*P* = 0.021, HR 4.175 (1.235-14.117) and *P* = 0.018, HR 4.366 (0.294-14.736)). In addition, N-cadherin low cases had a better DFS and OS (*P* = 0.015, HR 0.162 (0.037-0.705) and *P* = 0.014, HR 0.158 (0.036-0.687)). However, in the multivariate analysis, we found that E-cadherin low and N-cadherin high were not an independent adverse prognostic marker (*P* > 0.05) (Tables S1 and S2).

### 3.4. Correlations between the Expression of EMT Markers, E-Cadherin Low and N-Cadherin High, Pathological Features, and Stage of CRC

Table S3 shows the relationship of multiple clinicopathological factors with E-cadherin and N-cadherin. The expression of E-cadherin and N-cadherin was remarkably related to pathological TNM stage (I, II, III, and IV; *P* = 0.049 and *P* = 0.015), tumor size (<3 cm and ≥3; *P* = 0.004 and *P* = 0.028), depth grading of tumor invasion (T1+T2 and T3+T4; *P* = 0.001 and *P* = 0.002), and lymph node status (N0 and N1+N2; *P* = 0.016 and *P* = 0.030) (Table S3).

### 3.5. The Correlation between the Expressions of CDX2, E-Cadherin, and N-Cadherin

The relationships between the expressions of CDX2 and EMT markers in human CRC were evaluated. By the use of the Pearson correlation coefficient test, our group measured that enhanced expressions of CDX2 had significant relationships with E-cadherin (*P* = 0.01, *r* = 0.375) and N-cadherin (*P* = 0.002, *r* = −0.435) (Table S4).

### 3.6. Survival Analysis of Pathological Features and Serum Markers

We measured the Kaplan-Meier survival of RFS and OS for several clinical factors (Figures 2(g) and 3(d), Figure S1A-S1F, and Figure S2A-S2E). The results considered that the RFS was related to tumor size (*P* = 0.005), tumor differentiation (*P* = 0.042), T stage (*P* = 0.033), TNM stage (*P* = 0.023), lymph node metastasis (*P* = 0.017), M stage (*P* = 0.043), and preoperative CA199 level (*P* = 0.001). In multivariate assays, tumor differentiation (*P* = 0.023) and preoperative CA199 level (*P* < 0.001) were independent prognostic factor remarkably related with RFS (Table S1). In addition, univariate analyses found that the OS was correlated with tumor size (*P* = 0.003) T stage (*P* = 0.023), TNM stage (*P* = 0.006), lymph node metastasis (*P* = 0.010), M stage (*P* = 0.014), and preoperative CA199 level (*P* = 0.002) (Table S2). In multivariate assays, preoperative CA199 level (*P* = 0.002) was identified as independent prognostic factors (Table S2).

### 3.7. The Expression Status of CDX2 and CA199 in Combination Is Correlated with Prognosis of Patients with CRC

**Figure 4:**
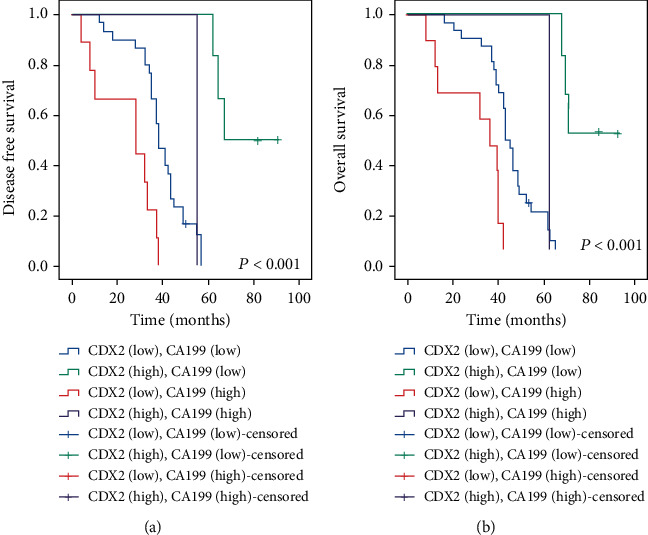
The expression status of CDX2 and CA199 in combination is associated with prognosis of patients with CRC. (a) Kaplan-Meier curves of RFS in patients with combinations of different levels of CDX2 and CA199 expressions. (b) Kaplan-Meier curves of OS in patients with combinations of different levels of CDX2 and CA199 expressions.

In multivariate assays, we found that CDX2 and CA199 expression levels were independent prognostic factor of DFS and OS (Tables S1 and S2). Therefore, we studied the relationship between CDX2 and CA199 in combination and prognosis of patients with CRC. Moreover, when the two examinations were analyzed in combination, patients with low expressions of CDX2 and high expressions of CA199 experienced a worse prognosis of OS and DFS, compared with low expressions of CDX2 and high expressions of CA199 or high expression level of CDX2 and low expression level of CA199 (log-rank test, *P* < 0.001) (Figures 4(a) and 4(b)). Therefore, the low expression of CDX2 and high expression of CA199 in combination is positively related with poor clinical outcome in CRC cases.

### 3.8. CDX2 Expression Is Related to Proliferation, Metastasis, and EMT of CRC Cells

**Figure 5:**
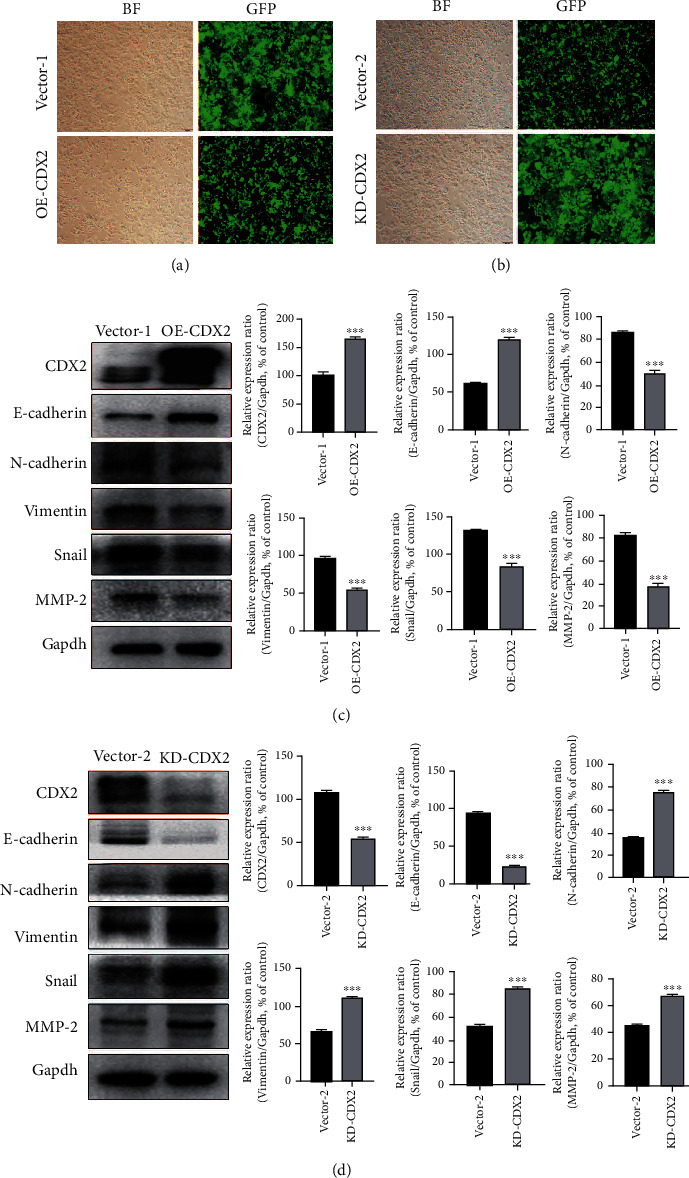
Overexpression of CDX2 suppressed EMT in colorectal cancer cells. (a, b) Lentiviral transduction efficiency was determined by a GFP fluorescence. (c) The upregulation of CDX2 in HCT-116 cells demonstrated by Western blot. (d) Knockdown of CDX2 in HCT-116 cells confirmed by Western blot, and Western blot showed reduced E-cadherin expression and enhanced N-cadherin, Vimentin, Snail, and MMP-2 expressions.

**Figure 6:**
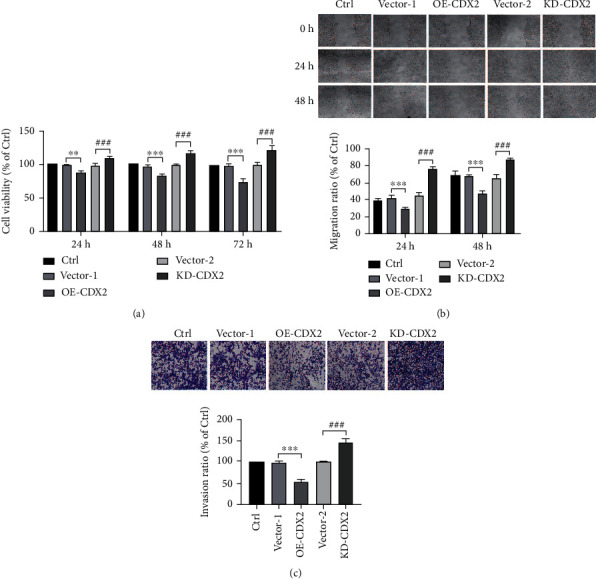
Overexpression of CDX2 inhibited proliferation, invasion, and metastasis in colorectal cancer cells. (a) Overexpression of CDX2 decreased the cell viability, while knockdown of CDX2 increased the cell viability by MTT assay. (b) Overexpression of CDX2 inhibited the migration of HCT-116 cells. (c) Overexpression of CDX2 reduced the invasion rate of HCT-116 cells while enhanced the invasion rate of HCT-116 cells by transwell assay.

To confirm that CDX2 influenced on CRC cells, we established stable cell lines from HCT-116 cells by overexpression (OE-CDX2) and knockdown (KD-CDX2) of CDX2. Successful overexpression and knockdown of CDX2 were demonstrated using the GFP signal (Figures 5(a) and 5(b)) and by Western blot (Figures 5(c) and 5(d)). OE-CDX2 remarkably elevated protein levels of E-cadherin and downregulated expression of N-cadherin, Vimentin, Snail1, and MMP family protein expression of MMP2 (Figure 5(c)). Conversely, KD-CDX2 significantly inhibited protein levels of E-cadherin and increased expression of N-cadherin, Vimentin, Snail1, and MMP family protein expression of MMP2 (Figure 5(d)). In addition, OE-CDX2 in HCT-116 cells decreased cell viability, while KD-CDX2 in HCT-116 cells increased in HCT-116 cells by MTT (Figure 6(a)). Moreover, OE-CDX2 significantly reduced the migratory and invasive capacities of HCT-116 cells, while KD-CDX2 remarkably enhanced the migratory and invasive capacities of HCT-116 cells by wound healing and transwell assays (Figures 6(b) and 6(c)). These results suggest that CDX2 promoted proliferation, invasion, and metastatic potential of CRC cells. Taken together, overexpression of CDX2 suppressed proliferative, invasive, migratory behaviors and EMT of CRC cells.

### 3.9. Correlation between CDX2 Expression and Immune Infiltrating Level in CRC

**Figure 7:**
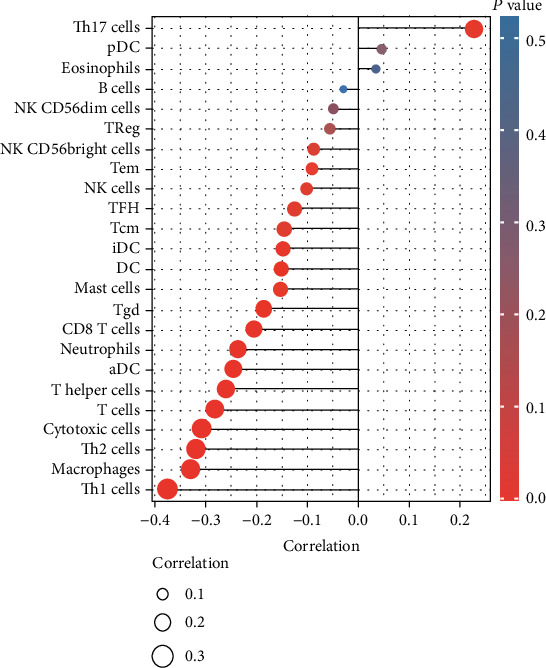
Correlation of CDX2 expression with immune infiltration level in CRC. The expression of CDX2 was negatively associated with Th1 cells, macrophages, Th2 cells, cytotoxic cells, T cells, and T helper cells.

Then, we explored the correlation between immune infiltration and CDX2 expression. As shown in Figure 7, we found that the expression of CDX2 was negatively associated with Th1 cells, macrophages, Th2 cells, cytotoxic cells, T cells, and T helper cells.

## 4. Discussion

CRC is one of the most prevalent forms of cancer in the world, and its definition describes it as a cancerous growth that originates in the epithelial tissue of the colon or rectum [24, 25]. The stage of cancer that is present at the time of diagnosis has a significant impact on whether or not a person can survive colorectal cancer. The five-year survival rate is approximately 90% for localized disease, 70% for regional disease, and only 13% for far metastatic CRC [26]. However, due to the huge magnitude of OS rates investigated across multiple phases, TNM-associated prognostic variables are unable to accurately predict the outcomes for patients [27]. At the same time, an individual's chance of cancer returning after surgery cannot be accurately anticipated due to the large amount of variation that exists across people [28]. Moreover, the clinical decision-making procedures would benefit from the identification of biomarkers that would allow doctors to differentiate between these cancers and those that have a high potential to metastasize. Increased levels of CEA, CA199, and Ki67 and decreased of E-cadherin level were related to poor OS [29]. Then, serum markers such as CEA and CA199 have also been used for the diagnosis of CRC and postoperative detection of therapeutic effect [30]. We discovered a link between tumor size, T stage, TNM stage, lymph node metastasis, M stage, and preoperative CA199 level, both of which were factors in determining the RFS and OS. The preoperative level of CA199 was found to be an independent prognostic factor after being subjected to a multivariate Cox regression analysis. In recent years, with the development of technology in molecular biology, the examination of cancer markers has become increasingly commonplace for the purposes of early cancer screening and diagnosis, directing treatment, monitoring cancer recurrence and metastasis, and estimating prognosis and survival. As a result, one of our goals is to identify a biomarker that has the highest level of connection with the CRC prognosis.

The transcription factor that is particular to the intestines CDX2 plays a vital role in maintaining the normal function of the colonic epithelium [31] and has been demonstrated to be a tumor suppressor [32, 33]. The morbidity of CDX2 loss was 19%, which is close to the results that were previously published on patients with stage IV CRC [12, 34]. Another study found that the level of CDX2 expression in CRC tissue samples was lower than in normal samples. In addition to this, a negative correlation can be shown between the expression of CDX2 and TNM staging, lymph node metastasis, and distant metastasis [11]. Then, the absence of CDX2 was found to have a significant correlation with indicators such MSI-H and BRAFmut [12, 34]. We hypothesized that CDX2 had a significant relationship with tumor size, the depth grading of tumor invasion, and the presence or absence of lymph nodes. Two recent retrospective researches have published CDX2 loss as a predictive marker for treatment advantage of chemotherapy in stage II [9] and stage III [12]. IHC analysis for CDX2 is used as a marker for intestinal differentiation in cancers of unknown origin in clinical diagnosis. This analysis is performed using immunohistochemistry. It is uncontrolled in some of the people who have CRC, and decreased expression of CDX2 has been linked to a bad prognosis in these patients [9, 11, 13, 35–37]. In a similar vein, some research revealed that a lower CDX2 was connected with a worse OS and RFS in patients who had CRC. Our research found that patients who had low levels of CDX2 expression had a significantly lower DFS and OS than patients who had high levels of CDX2 expression when analyzed using the univariate Cox regression method. In addition to this, CDX2 was shown to be an independent prognostic predictor of OS but not RFS. In general, CDX2 has the potential to be an important biomarker for directing assessment of the course of tumors and their prognoses [38, 39]. However, Tarazona et al. suggested that CDX2-negative tumors were correlated with shorter DFS [40]. In multivariate assays, we discovered that CDX2-low was an independent adverse prognostic marker of OS and DFS. Therefore, we studied the relationship between CDX2 and CA199 in combination and prognosis of patients with CRC. The result found that the low expressions of CDX2 and high expressions of CA199 in combination are positively related with poor outcomes in CRC patients.

Although EMT is not limited to cancer cells, it is often abnormally regulated in cancer cells. Cellular plasticity is required for EMT to occur [17, 41, 42]. After EMT has been induced, E-cadherin expression is downregulated, and epithelial cells lose their characteristic cobblestone appearance and become more round. The cells acquire a mesenchymal morphology, with a spindle form, and exhibit biomarker characteristic of mesenchymal cells, most notably N-cadherin, vimentin, and fibronectin [43]. In CRC, low expression of E-cadherin is deemed as independent prognostic factors of enhanced survival [44]. Furthermore, according to EMT markers, low level of E-cadherin [45] and high level of Vimentin, N-cadherin [46], and Slug have been associated with poorer prognosis in CRC. The univariate analysis revealed that E-cadherin low cases had a worse DFS and OS and N-cadherin low cases had a better DFS and OS. However, in the multivariate analysis, we found that E-cadherin low and N-cadherin high were not an independent adverse prognostic marker. And the levels of E-cadherin and N-cadherin were remarkably associated with pathological TNM stage, tumor size, depth grading of tumor invasion, and lymph node status. A mass of evidences considers an important role for CDX2 as a tumor suppressor in CRC. However, the precise functions of CDX2 involved in EMT progress in CRC remain to be illuminated.

Zheng et al. and Yu et al. found that restoration of CDX2 expression level significantly inhibited the aggressive phenotype of colon cancer cells, such as viability, invasive and migratory abilities, and colony formation [47, 48]. The decrease of CDX2 has been considered to be a progenitor for metastatic colon cancer to execute EMT [49]. Through the use of in vitro and in vivo experiments as well as a collection of samples from CRC patients, researchers were able to determine that CDX2 is a significant inhibitor of the invasion phenotype and EMT in colon cancer. And they found that CDX2 was positively associated E-cadherin expression and was negatively related with Snail and vimentin expressions in clinical CRC samples [38]. We also confirmed that the protein levels of high CDX2 had significant positive correlation with E-cadherin. Besides, we discovered that the levels of CDX2, E-cadherin, and N-cadherin had a remarkable association with tumor size, depth grading of tumor invasion, and lymph node status. This is significant when taking into consideration the fact that CDX2 and EMT markers may play a role in the growth, invasion, and metastasis of tumors. Therefore, we established stable cell lines from HCT-116 cells by OE-CDX2 and KD-CDX2 to confirm the results. We found that OE-CDX2 suppressed proliferative, invasive, migratory behaviors of CRC cells through inhibiting EMT, while KD-CDX2 activated proliferative, invasive, migratory behaviors by promoting EMT.

Tumor-infiltrating immune cells (TIICs) are an essential component of the intricate microenvironment that controls the onset and course of a wide variety of malignancies. The number of lymphocytes that infiltrate a tumor and their activity level are two of the most critical factors that can be used to predict how long a patient will live with cancer. Then, we explored the correlation between immune infiltration and CDX2 expression and found that the expression of CDX2 was negatively associated with Th1 cells, macrophages, Th2 cells, cytotoxic cells, T cells, and T helper cells. Our findings suggested that CDX2 may be a potential biomarker for tumor immunotherapy response.

However, our present study has some limitations. Firstly, considering the limited size of the sample, it will be necessary to do extensive clinical tests. Secondly, we just performed in in vitro to explore the function of CDX2. More in vivo experiments were needed to further confirm our findings.

## 5. Conclusion

Our findings consider that CDX2 loss is an independent risk factor of adverse OS and DFS. The CDX2 remarkably correlated with the aggressive behavior and significantly associated with the EMT markers. And the low level of CDX2 and high level of CA199 in combination are positively related with poor prognosis in patients with CRC. Besides, our data confirmed that CDX2 inhibited proliferation and metastasis through inhibiting EMT.

## Data Availability

The datasets used and/or analyzed during the current study are available from the corresponding author on reasonable request.

## Supplementary Materials

Supplementary 1 Figure S1: Kaplan-Meier survival curves revealed RFS of clinicopathological factors in CRC. (A) Tumor size. (B) Tumor differentiation. (C) T stage. (D) TNM stage. (E) Lymph node status. (F) M stage. The *P* value was obtained using the log-rank test of the differences.

Supplementary 2 Figure S2: Kaplan-Meier survival curves exhibited OS of clinical and pathologic dates in CRC. (A) Tumor size. (B) T stage. (C) TNM stage. (D) Lymph node status. (E) M stage. The *P* value was obtained using the log-rank test of the differences.

Supplementary 3 Table S1: Univariate and multivariate Cox proportional hazard analysis of DFS for patients with CRC.

Supplementary 4 Table S2: Univariate and multivariate Cox proportional hazard analysis of OS for patients with CRC.

Supplementary 5 Table S3: Expression of CDX2, N-cadherin, and E-cadherin protein and clinicopathological parameters in colorectal cancer tissues.

Supplementary 6 Table S4: Association of CDX2 protein expression with N-cadherin protein expression and E-cadherin protein expression.
