# Supplementary material for: CDX2 as a Predictive Biomarker Involved in Immunotherapy Response Suppresses Metastasis through EMT in Colorectal Cancer
Source: Dis Markers. 2022 Oct 12;2022:9025668. doi: 10.1155/2022/9025668 (PMC9582897; doi:10.1155/2022/9025668)
Supplement: Supplementary 1 — Figure S1: Kaplan-Meier survival curves revealed RFS of clinicopathological factors in CRC. (A) Tumor size. (B) Tumor differentiation. (C) T stage. (D) TNM stage. (E) Lymph node status. (F) M stage. The P value was obtained using the log-rank test of the differences. [file 9025668.f1.pdf]

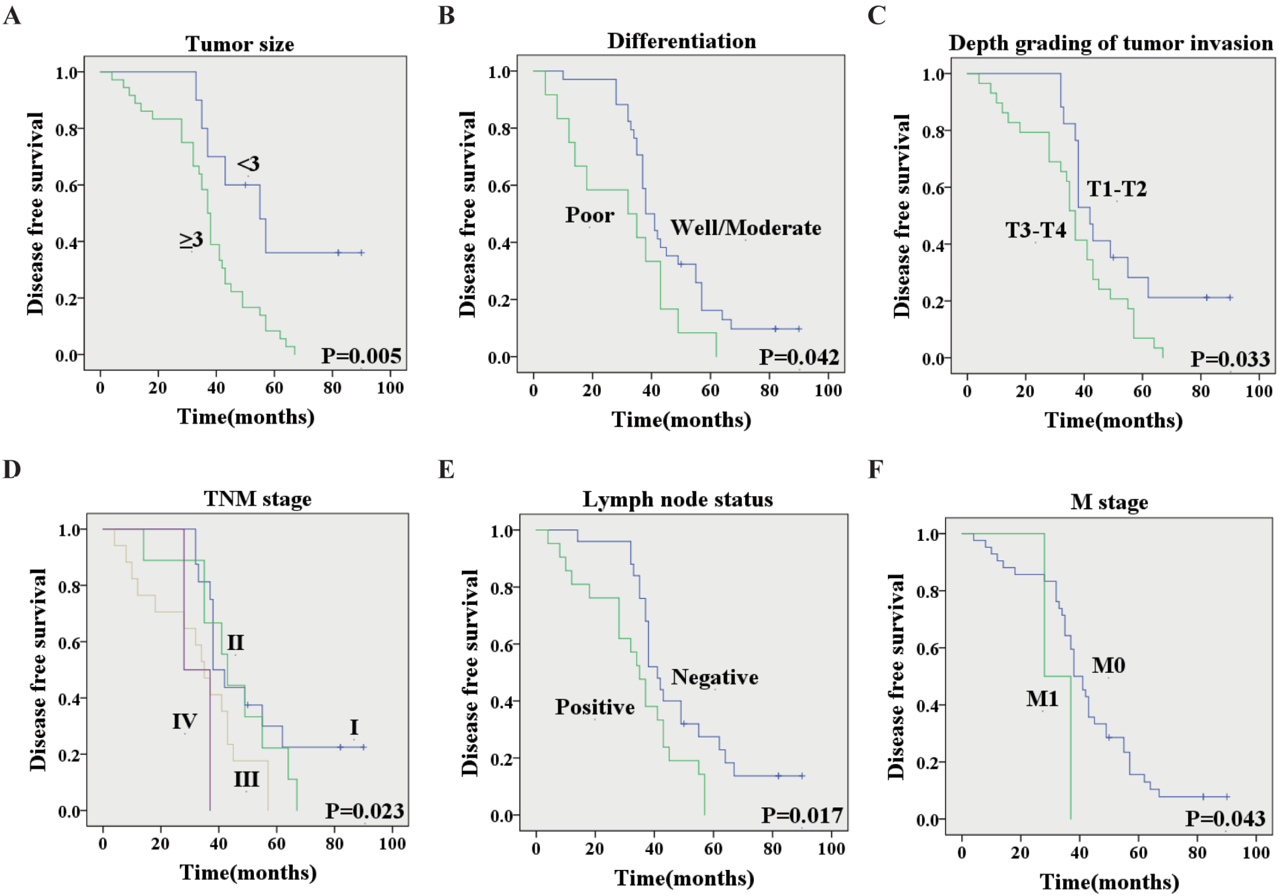

A
Tumor size
Disease free survival
Time(months)
<3
≥3
P=0.005
B
Differentiation
Disease free survival
Time(months)
Poor
Well/Moderate
P=0.042
C
Depth grading of tumor invasion
Disease free survival
Time(months)
T1-T2
T3-T4
P=0.033
D
TNM stage
Disease free survival
Time(months)
I
II
III
IV
P=0.023
E
Lymph node status
Disease free survival
Time(months)
Negative
Positive
P=0.017
F
M stage
Disease free survival
Time(months)
M0
M1
P=0.043
