# Supplementary figures and images for: CDX2 as a Predictive Biomarker Involved in Immunotherapy Response Suppresses Metastasis through EMT in Colorectal Cancer
Source: Dis Markers. 2022 Oct 12;2022:9025668. doi: 10.1155/2022/9025668 (PMC9582897; doi:10.1155/2022/9025668)

A

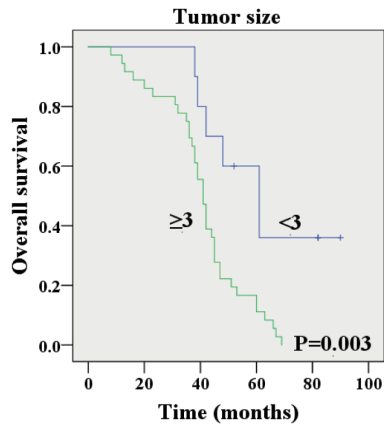

B

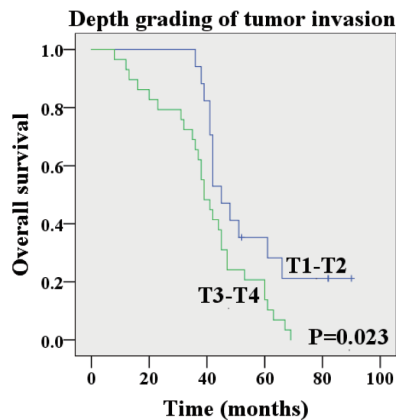

C

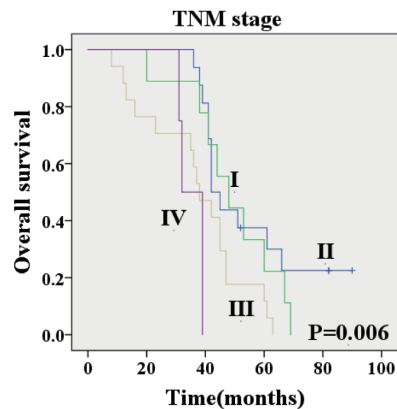

D

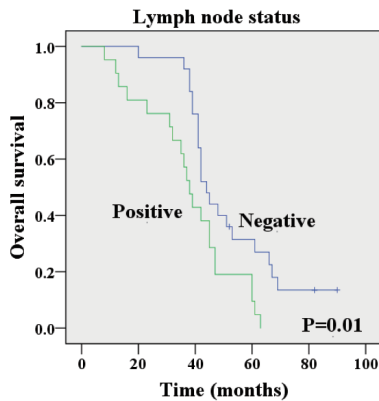

E

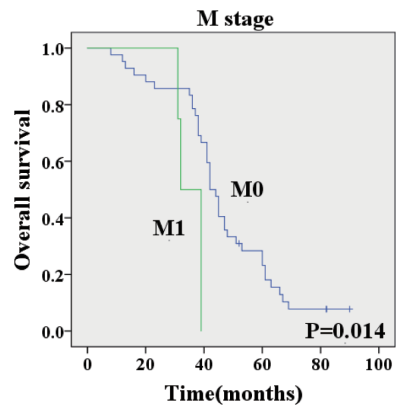

Supplement: Supplementary 2 — Figure S2: Kaplan-Meier survival curves exhibited OS of clinical and pathologic dates in CRC. (A) Tumor size. (B) T stage. (C) TNM stage. (D) Lymph node status. (E) M stage. The P value was obtained using the log-rank test of the differences. [file 9025668.f2.pdf]
