# Supplementary material for: CDX2 as a Predictive Biomarker Involved in Immunotherapy Response Suppresses Metastasis through EMT in Colorectal Cancer
Source: Dis Markers. 2022 Oct 12;2022:9025668. doi: 10.1155/2022/9025668 (PMC9582897; doi:10.1155/2022/9025668)
Supplement: Supplementary 3 — Table S1: Univariate and multivariate Cox proportional hazard analysis of DFS for patients with CRC. [file 9025668.f3.docx]

Table S1 Univariate and multivariate Cox proportional hazards analysis of DFS for patients with CRC

| Variables | Univariate analysis HR (95% CI) | *p*-value | Multivariate analysis HR(95% CI) | *p*-value |
| --- | --- | --- | --- | --- |
| Age |  | 0.524 |  |  |
| ≤60 |  |  |  |  |
| >60 |  |  |  |  |
| Sex |  | 0.380 |  |  |
| Male |  |  |  |  |
| Female |  |  |  |  |
| Tumor location |  | 0.616 |  |  |
| colon |  |  |  |  |
| rectal |  |  |  |  |
| Tumor size(cm) | 0.310（0.128-0.750） | 0.009 |  |  |
| <3 |  |  |  |  |
| ≥3 |  |  |  |  |
| Differentiation | 0.511（0.258-1.010） | 0.053 | 0.437（0.214-0.890） |  |
| Well/moderate |  |  |  | 0.023 |
| Poor/undifferentiated |  |  |  |  |
| T stage | 0.506（0.261-0.980） | 0.044 |  |  |
| T1-T2 |  |  |  |  |
| T3-T4 |  |  |  |  |
| TNM stage | 0.226（0.068-0.751） | 0.030 |  |  |
| Ⅰ |  |  |  |  |
| Ⅱ |  |  |  |  |
| Ⅲ |  |  |  |  |
| Ⅳ |  |  |  |  |
| Lymph node metastasis | 0.486（0.258-0.915） | 0.025 |  |  |
| No |  |  |  |  |
| Yes |  |  |  |  |
| Distant metastasis | 0.348（0.116-1.038） | 0.058 |  |  |
| No |  |  |  |  |
| Yes |  |  |  |  |
| Preoperative CEA level （ng/mL） |  | 0.089 |  |  |
| ≤5 |  |  |  |  |
| >5 |  |  |  |  |
| Preoperative CA199 level （U/mL） | 0.309（0.146-0.651） | 0.002 | 0.183(0.079-0.423) |  |
| <37 |  |  |  | <0.001 |
| ≥37 |  |  |  |  |
| CDX2 protein expression | 21.475（2.884-159.885） | 0.003 | 28.068（3.699-212.960） |  |
| Low |  |  |  | 0.001 |
| High |  |  |  |  |
| N-cadherin protein expression | 0.162（0.037-0.705） | 0.015 |  |  |
| Low |  |  |  |  |
| High |  |  |  |  |
| E-cadherin protein expression | 4.175（1.235-14.117） | 0.021 |  |  |
| Low |  |  |  |  |
| High |  |  |  |  |
