# Supplementary material for: CDX2 as a Predictive Biomarker Involved in Immunotherapy Response Suppresses Metastasis through EMT in Colorectal Cancer
Source: Dis Markers. 2022 Oct 12;2022:9025668. doi: 10.1155/2022/9025668 (PMC9582897; doi:10.1155/2022/9025668)
Supplement: Supplementary 4 — Table S2: Univariate and multivariate Cox proportional hazard analysis of OS for patients with CRC. [file 9025668.f4.docx]

Table S2 Univariate and multivariate Cox proportional hazards analysis of OS for patients with CRC

| Variables | Univariate analysis HR (95% CI) | *p*-value | Multivariate analysis HR(95% CI) | *p*-value |
| --- | --- | --- | --- | --- |
| Age |  |  |  |  |
| ≤60 |  | 0.477 |  |  |
| >60 |  |  |  |  |
| Sex |  | 0.389 |  |  |
| Male |  |  |  |  |
| Female |  |  |  |  |
| Tumor location |  | 0.767 |  |  |
| colon |  |  |  |  |
| rectal |  |  |  |  |
| Tumor size(cm) | 0.290（0.120-0.703） | 0.006 |  |  |
| <3 |  |  |  |  |
| ≥3 |  |  |  |  |
| Differentiation |  | 0.067 |  |  |
| Well/moderate |  |  |  |  |
| Poor/undifferentiated |  |  |  |  |
| T stage | 0.481（0.248-0.932） | 0.030 |  |  |
| T1-T2 |  |  |  |  |
| T3-T4 |  |  |  |  |
| TNM stage | 0.172（0.050-0.586） | 0.005 |  |  |
| Ⅰ |  |  |  |  |
| Ⅱ |  |  |  |  |
| Ⅲ |  |  |  |  |
| Ⅳ |  |  |  |  |
| Lymph node metastasis | 0.452（0.239-0.854） | 0.014 |  |  |
| No |  |  |  |  |
| Yes |  |  |  |  |
| Distant metastasis | 0.274（0.090-0.837） | 0.023 |  |  |
| No |  |  |  |  |
| Yes |  |  |  |  |
| Preoperative CEA level （ng/mL） |  | 0.070 |  |  |
| ≤5 |  |  |  |  |
| >5 |  |  |  |  |
| Preoperative CA199 level （U/mL） | 0.327（0.156-0.688） | 0.003 | 0.186(0.079-0.441) |  |
| <37 |  |  |  | <0.001 |
| ≥37 |  |  |  |  |
| CDX2 protein expression | 27.691（3.614-212.163） | 0.001 | 38.902(4.979-303.962) |  |
| Low |  |  |  | <0.001 |
| High |  |  |  |  |
| N-cadherin protein expression | 0.158（0.036-0.687） | 0.014 |  |  |
| Low |  |  |  |  |
| High |  |  |  |  |
| E-cadherin protein expression | 4.366（1.294-14.736） | 0.018 |  |  |
| Low |  |  |  |  |
| High |  |  |  |  |
