# Supplementary material for: CDX2 as a Predictive Biomarker Involved in Immunotherapy Response Suppresses Metastasis through EMT in Colorectal Cancer
Source: Dis Markers. 2022 Oct 12;2022:9025668. doi: 10.1155/2022/9025668 (PMC9582897; doi:10.1155/2022/9025668)
Supplement: Supplementary 5 — Table S3: Expression of CDX2, N-cadherin, and E-cadherin protein and clinicopathological parameters in colorectal cancer tissues. [file 9025668.f5.docx]

Table S3 Expression of CDX2, N-cadherin and E-cadherin protein and clinicopathological parameters in colorectal cancer tissues

| Variables | CDX2 | | *p*-value | N-cadherin | | *p*-value | E-cadherin | | *p*-value |
| --- | --- | --- | --- | --- | --- | --- | --- | --- | --- |
|  | High | Low |  | High | Low |  | High | Low |  |
| Age |  |  |  |  |  |  |  |  |  |
| ≤60 | 2 | 18 | 0.388 | 18 | 2 | 0.868 | 2 | 18 | 0.591 |
| >60 | 5 | 21 |  | 23 | 3 |  | 4 | 22 |  |
| Sex |  |  |  |  |  |  |  |  |  |
| Male | 4 | 24 | 0.826 | 26 | 2 | 0.311 | 2 | 26 | 0.138 |
| Female | 3 | 15 |  | 15 | 3 |  | 4 | 14 |  |
| Tumor location |  |  |  |  |  |  |  |  |  |
| colon | 2 | 17 | 0.457 | 18 | 1 | 0.305 | 1 | 18 | 0.189 |
| rectal | 5 | 22 |  | 23 | 4 |  | 5 | 22 |  |
| Tumor size(cm) |  |  |  |  |  |  |  |  |  |
| <3 | 4 | 6 | 0.014 | 7 | 3 | 0.028 | 4 | 6 | 0.004 |
| ≥3 | 3 | 33 |  | 34 | 2 |  | 2 | 34 |  |
| Differentiation |  |  |  |  |  |  |  |  |  |
| Well/moderate | 6 | 28 | 0.440 | 30 | 4 | 0.743 | 4 | 30 | 0.665 |
| Poor | 1 | 11 |  | 11 | 1 |  | 2 | 10 |  |
| T stage |  |  |  |  |  |  |  |  |  |
| T1-T2 | 5 | 12 | 0.040 | 12 | 5 | 0.002 | 6 | 11 | 0.001 |
| T3-t4 | 2 | 27 |  | 29 | 0 |  | 0 | 29 |  |
| TNM stage |  |  |  |  |  |  |  |  |  |
| Ⅰ | 5 | 11 | 0.063 | 11 | 5 | 0.015 | 5 | 11 | 0.049 |
| Ⅱ | 2 | 7 |  | 9 | 0 |  | 1 | 8 |  |
| Ⅲ | 0 | 17 |  | 17 | 0 |  | 0 | 17 |  |
| Ⅳ | 0 | 4 |  | 4 | 0 |  | 0 | 4 |  |
| Lymph node metastasis |  |  |  |  |  |  |  |  |  |
| No | 7 | 18 | 0.008 | 20 | 5 | 0.030 | 6 | 19 | 0.016 |
| Yes | 0 | 21 |  | 21 | 0 |  | 0 | 21 |  |
| Distant metastasis |  |  |  |  |  |  |  |  |  |
| No | 7 | 35 | 0.375 | 37 | 5 | 0.465 | 6 | 36 | 0.418 |
| Yes | 0 | 4 |  | 4 | 0 |  | 0 | 4 |  |
| Preoperative CEA level（ng/mL） |  |  |  |  |  |  |  |  |  |
| ≤5 | 6 | 26 | 0.313 | 27 | 5 | 0.117 | 4 | 28 | 0.869 |
| >5 | 1 | 13 |  | 14 | 0 |  | 2 | 12 |  |
| Preoperative CA199 level （U/mL） |  |  |  |  |  |  |  |  |  |
| <37 | 6 | 30 | 0.604 | 31 | 5 | 0.212 | 5 | 31 | 0.747 |
| ≥37 | 1 | 9 |  | 10 | 0 |  | 1 | 9 |  |
