# Supplementary material for: CDX2 as a Predictive Biomarker Involved in Immunotherapy Response Suppresses Metastasis through EMT in Colorectal Cancer
Source: Dis Markers. 2022 Oct 12;2022:9025668. doi: 10.1155/2022/9025668 (PMC9582897; doi:10.1155/2022/9025668)
Supplement: Supplementary 6 — Table S4: Association of CDX2 protein expression with N-cadherin protein expression and E-cadherin protein expression. [file 9025668.f6.docx]

Table S4 Association of CDX2 protein expression with N-cadherin protein expression and E-cadherin protein expression.

|  | CDX2 |  |  |  |
| --- | --- | --- | --- | --- |
| Characteristic | Low | High | *p*-value | Rho^a^ |
| N-cadherin |  |  |  |  |
| Low | 2 | 37 | 0.002 | -0.435 |
| High | 3 | 4 |  |  |
| E-cadherin |  |  |  |  |
| Low | 36 | 3 | 0.010 | 0.375 |
| High | 4 | 3 |  |  |

^a^Spearman rank test.
